# Use of primary oral vancomycin prophylaxis to stem an outbreak of *Clostridioides difficile* infection in intensive care patients

**DOI:** 10.1017/ash.2025.10095

**Published:** 2025-09-18

**Authors:** Anjali Kewalramani, Shaurya Sharma, Katrina Sandejas, Daniel Rampersad, Qu Zhong, Briana Episcopia, Leon Boudourakis, John Quale

**Affiliations:** 1 Department of Medicine, NYC Health+Hospitals/Kings County, Brooklyn, NY, USA; 2 Department of Pharmacy, NYC Health+Hospitals/Kings County, Brooklyn, NY, USA; 3 Department of Infection Prevention, NYC Health+Hospitals/Kings County, Brooklyn, NY, USA; 4 Department of Surgery (Boudourakis), NYC Health+Hospitals/Kings County, Brooklyn, NY, USA

## Abstract

Over 5.5-months, hospital-onset *Clostridioides difficile* infections (HO-CDI) in intensive care units (ICUs) increased from a baseline 6.2 cases to 19.1 cases per 10,000 patient-days (*P* = .03). Primary oral vancomycin prophylaxis (OVP) to select patients was initiated; subsequently there were 5.7 cases /10,000 patient-days (*P* = .05). Primary OVP curbed an outbreak of HO-CDI.

## Introduction

Traditional modalities to limit hospital-onset *Clostridioides difficile* infections (HO-CDI) emphasize antimicrobial stewardship and infection control measures. “High-risk” antibiotics for HO-CDI include clindamycin, fluoroquinolones, and broad-spectrum β-lactams. Infection control measures emphasize hand hygiene and environmental decontamination. However, risk of CDI in patients in intensive care units (ICUs) may be more related to asymptomatic carriage upon admission with subsequent expression, and not from person-to-person acquisition during hospitalization.^1^

While the use of oral vancomycin prophylaxis (OVP) has been shown to reduce CDI recurrences, the use of primary OVP is more controversial.^2,3^ Several reports have documented significant reductions in cases of CDI with the use of primary OVP.^4–6^ We document utility of primary OVP during a sustained outbreak of HO-CDI in ICUs in a large urban tertiary care hospital.

## Methods

NYC Health + Hospitals/Kings County is a 627-bed, level 1 trauma center, public safety net hospital in Brooklyn, NY. A 20-bed medical ICU adjoins a 20-bed surgical/neurosurgical ICU. *C. difficile* testing included an initial GDH and toxin assay, followed by toxin PCR for discrepant results. Incident cases of HO-CDI were reviewed. Three periods were examined. The Baseline Period was from January 1, 2023, to September 30, 2023. From October 1, 2023, to March 15, 2024 a sustained rise in HO-CDI cases occurred in the ICUs (Outbreak Period). An intervention period was from March 16, 2024, to August 31, 2024.

The intervention involving primary OVP was developed and implemented by members of the Departments of Pharmacy, ICU, Infection Prevention, and Infectious Diseases. Based on prior studies and our review of preceding cases at out hospital, the intervention targeted patients age ≥60 years who were hospitalized for ≥ 7 days (or recently hospitalized within 30 d) and were receiving high-risk systemic antibiotics: piperacillin-tazobactam, cephalosporins (ceftriaxone and cefepime), fluoroquinolones (levofloxacin), and/or clindamycin.^4,5^ Patients receiving only perioperative prophylactic antibiotics, those receiving metronidazole, or those unable to tolerate oral medications were not given primary OVP. Dosing was 125 mg once daily; twice daily administration was allowed for severely immunocompromised patients. OVP was continued for up to five days postcompletion of systemic antibiotic therapy.

Measured variables during each period included the number and rate of HO-CDI, utilization of high-risk antibiotics, hand-hygiene compliance rates, and the number and rate of patients with positive cultures for vancomycin-resistant *E. faecalis* and *E. faecium* (VRE). Statistical comparison of rates was made using the Test based methodology and X^2^ analysis.^7^

## Results

### Baseline period

During the Baseline Period, there were five HO-CDI in the ICUs (rate 6.2 cases/10,000 patient-days; Table 1). The average age of the five patients was 63.2 ± 21 years and the duration of hospitalization was 10.0 ± 3.5 days; all were hospitalized for ≥ seven days. All five received a broad-spectrum β-lactam prior to the onset of *C. difficile*. Antibiotic usage for broad-spectrum antibiotics used in the ICUs is given in the Table 1. There were 12 patients in the ICUs with cultures with VRE (rate 15.0 cases per 10,000 patient-days). Hand hygiene compliance was 67.8% (of 3,159 observations).

**Table 1.** Frequency of *C. difficile*, vancomycin resistant enterococci, and antibiotic usage of high-risk antibiotics during the baseline, outbreak, and intervention periods

	Baseline	Outbreak	Intervention
HO-*C. difficile* (Cases per 10,000 patient-days)			
ICUs	6.2^*^	19.1	5.7^*^
Non-ICUs	1.6	2.0	2.9
Vancomycin-resistant enterococci (Cases per 10,000 patient-days)			
ICUs	15.0	7.0	14.2
Non-ICUs	3.8	4.2	4.7
ICU antibiotic usage (Days of treatment/1,000 patient-days)			
Cefepime	7.54	7.95	4.74^*^
Ceftriaxone	11.14	11.34	10.86
Levofloxacin	2.23^*^	1.03	2.27^*^
Meropenem	8.26^*^	7.01	7.62
Piperacillin/tazobactam	19.45	19.75	19.95

* *P* ≤ .05 compared to outbreak period.

### Outbreak period

During the Outbreak Period there were 11 HO-CDI in the ICUs (rate 19.1 cases/10,000 patient-days, *P* = .03 compared to the Baseline Period); the remainder of the hospital remained unchanged. The average age of the ICU patients with HO-CDI was 69.7 ± 14.9 years; all but one was >60 years of age. Patients were hospitalized for an average of 21.1 ± 17.8 days prior to infection; all were hospitalized for >7 days. Receipt of β-lactams was implicated in all cases: all 11 received piperacillin-tazobactam, one also recently received cefazolin, two cefepime, and one meropenem.

**Figure 1.**
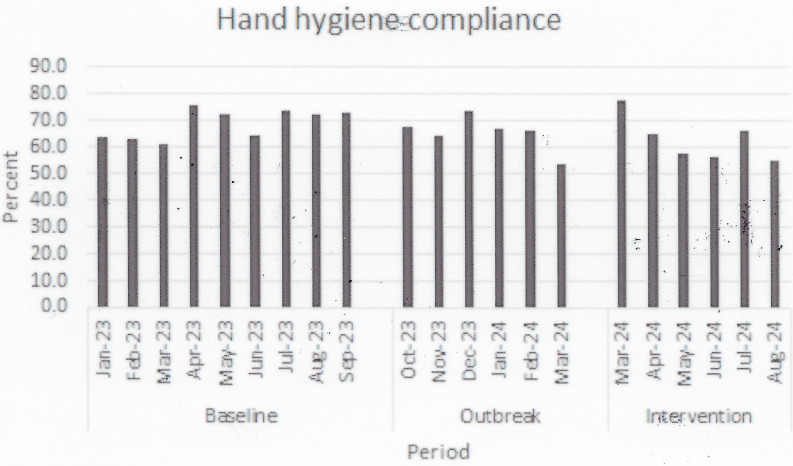
Monthly hand hygiene compliance rates in the ICUs during the three periods.

Compared to the Baseline Period, overall usage of important antibiotics did not change appreciably (Table 1) during the Outbreak Period (48.6–47.1 DOT/100 patient days), although levofloxacin and meropenem use decreased (Table 1). Oral vancomycin usage increased compared to the Baseline Period, from 1.14 to 3.29 DOT/100 patient days (*P* = <.001). During the Outbreak Period there were four patients in the ICUs with VRE, a rate similar to the Baseline Period. Hand hygiene compliance was also unchanged (65.0% of 2,094 observations, Figure 1).

### Intervention period

A focused strategy of primary OVP was developed based on (1) our patients’ demographic data gathered during our outbreak period and (2) a previously published study.^4^ During the Intervention Period, there were three cases of HO-CDI in the ICUs, a rate significantly less than that observed during the outbreak period (19.1 cases to 5.7 cases/10,000 patient-days, *P* = .048). The rate of patients with toxin-positive HO-CDI also fell, from 8.7 to 0 cases/10,000 patient-days (*P* = .03). While overall antibiotic use did not change, during the Intervention Period there was a significant decline in the use of cefepime and an increase in the use of levofloxacin (Table 1). As anticipated, oral vancomycin use increased significantly from the Outbreak to Intervention Periods (3.29–7.46 DOT per 100 patient-days, *P* < .001). During the Intervention Period the number of ICU patients with VRE remained unchanged. Finally, hand hygiene rates remained unchanged during the intervention period (61.9% of 2,049 observations).

## Discussion

While there is considerable data supporting the use of secondary OVP against *C. difficile*, there is relatively scant data about using vancomycin for primary prophylaxis.^2,3^ Two retrospective studies found reduced rates of *C. difficile* during periods of primary OVP among two populations: elderly patients on an infectious diseases ward and hematopoietic stem cell recipients.^5,6^ A third randomized controlled study also found significantly reduced rates of HO-CDI for high-risk elderly patients that received primary OVP.^4^ In our report, also emphasizing high-risk elderly patients, we found primary OVP was associated with a significant decrease in HO-CDI cases in intensive care areas.

The use of primary OVP may not be without consequences, however. Vancomycin resistance (defined as an MIC > 2 µg/ml) in *C. difficile* may be increasing.^8,9^ Despite fecal vancomycin concentrations that far exceed the MICs of resistant *C. difficile*, isolates with elevated MICs are associated with decreased 14-day initial cure rates and 30-day sustained clinical responses.^8^ Finally, use of secondary OVP has been associated with increased colonization with VRE.^10^ In our report we did have a non-significant increase in patients with VRE in the ICUs during the period of primary OVP. Because of these concerns, our Intervention Period was halted after 5.5 months.

Our investigation suffers from the limitations expected from a respective cohort study, including variability in risk factors during the time periods. For example, one confounding variable was the decreased usage of cefepime during the intervention period compared to the outbreak period. However, among the five high risk antibiotics examined in this study, cefepime accounted for ∼ 10–15% of antibiotic consumption, and the decrease in cefepime usage was offset by an increase in levofloxacin usage. In addition, the impact on VRE colonization may not have been fully appreciated, as detection may be delayed and dedicated screening measures were not implemented. Another confounding variable was the relatively low hand hygiene compliance rates throughout the study periods, certainly an area with room for improvement.

In summary, we found primary OVP in a targeted population of patients in our ICUs to be an effective modality in terminating an outbreak of *C. difficile* infection. Because of an increasing trend in the number of patients with VRE, and the potential impact on vancomycin susceptibility of *C. difficile*, the intervention was halted after a 5.5-month period.
